# Association of secondary displacement of distal radius fractures with cortical bone quality at the distal radius

**DOI:** 10.1007/s00402-020-03658-2

**Published:** 2020-10-31

**Authors:** A. M. Daniels, H. M. J. Janzing, C. E. Wyers, B. van Rietbergen, L. Vranken, R. Y. Van der Velde, P . P. M. M. Geusens, S. Kaarsemaker, M. Poeze, J. P. Van den Bergh

**Affiliations:** 1 Department of Surgery, VieCuri Medical Centre, Tegelseweg 210, 5912 BL Venlo, The Netherlands; 2 NUTRIM School for Nutrition and Translational Research in Metabolism, Maastricht University, Universiteitssingel 40, 6229 ER Maastricht, The Netherlands; 3 Department of Internal Medicine, Subdivision of Endocrinology, VieCuri Medical Centre, Tegelseweg 210, 5912 BL Venlo, The Netherlands; 4 Department of Internal Medicine, Maastricht University Medical Centre, P. Debyelaan 25, 6229 HX Maastricht, The Netherlands; 5 Orthopaedic Biomechanics, Department of Biomedical Engineering, Eindhoven University of Technology, De Rondom 70, 5612 AP Eindhoven, The Netherlands; 6 Department of Orthopaedic Surgery, Research School CAPHRI, Maastricht University, Universiteitssingel 40, 6229 ER Maastricht, The Netherlands; 7 Faculty of Medicine, Hasselt University, Martelarenlaan 42, 3500 Hasselt, Belgium; 8 Department of Orthopaedic Surgery, VieCuri Medical Centre, Tegelseweg 210, 5912 BL Venlo, The Netherlands; 9 Department of Surgery, Subdivision of Traumatology, Maastricht University Medical Centre, P. Debyelaan 25, 6229 HX Maastricht, The Netherlands

**Keywords:** Distal radius fracture (DRF), Fracture displacement, High-resolution peripheral quantitative CT (HR-pQCT), Bone microarchitecture and strength, Primary reduction

## Abstract

**Introduction:**

The aim of this study was to investigate the associations of patient characteristics, bone mineral density (BMD), bone microarchitecture and calculated bone strength with secondary displacement of a DRF based on radiographic alignment parameters.

**Materials and methods:**

Dorsal angulation, radial inclination and ulnar variance were assessed on conventional radiographs of a cohort of 251 patients, 38 men and 213 women, to determine the anatomic position of the DRF at presentation (primary position) and during follow-up.

Secondary fracture displacement was assessed in the non-operatively treated patients (*N* = 154) with an acceptable position, preceded (*N* = 97) or not preceded (*N* = 57) by primary reduction (baseline position). Additionally, bone microarchitecture and calculated bone strength at the contralateral distal radius and tibia were assessed by HR-pQCT in a subset of, respectively, 63 and 71 patients.

**Outcome:**

Characteristics of patients with and without secondary fracture displacement did not differ. In the model with adjustment for primary reduction [OR 22.00 (2.27–212.86), *p* = 0.008], total [OR 0.16 (95% CI 0.04–0.68), *p* = 0.013] and cortical [OR 0.19 (95% CI 0.05–0.80], *p* = 0.024] volumetric BMD (vBMD) and cortical thickness [OR 0.13 (95% CI 0.02–0.74), *p* = 0.021] at the distal radius were associated with secondary DRF displacement. No associations were found for other patient characteristics, such as age gender, BMD or prevalent vertebral fractures.

**Conclusions:**

In conclusion, our study indicates that besides primary reduction, cortical bone quality may be important for the risk of secondary displacement of DRFs.

**Electronic supplementary material:**

The online version of this article (10.1007/s00402-020-03658-2) contains supplementary material, which is available to authorized users.

## Introduction

Standard initial management for distal radius fractures (DRFs) at the emergency department (ED) is cast immobilisation (CI) preceded by closed reduction in the case of a dislocated fracture. Further management depends on the anatomic position, DRFs with acceptable position can be managed non-operatively [1]. Dislocated intra-articular DRFs often require surgical fixation to restore and retain correct fracture position. Patient characteristics, such as age and comorbidities, must be taken into account when making this decision [2–4]*.* It is of great importance to identify fractures at risk for displacement to achieve the most adequate treatment. By being able to anticipate early-stage instability, unnecessary manipulation can be prevented, surgical treatment options can be discussed timely and a reduction in complications such as mal-union might be accomplished.

In 1989, five risk factors for DRF instability were identified by Lafontaine et al. namely primary dorsal angulation exceeding 20 degrees, dorsal comminution, involvement of the radio-carpal joint, styloid ulnae fracture and patients aged over 60 years [5]. From that time on, many prediction rules for instability have been developed, some previous risk factors could not be confirmed by new studies and new risk factors for secondary displacement, such as radial shortening, have been identified [6–11]. In this debate, little attention was paid to the influence of bone properties such as bone mineral density (BMD), bone microarchitecture and calculated bone strength. BMD can be assessed using bone densitometry, whereas bone microarchitecture and separate assessment of trabecular and cortical bone requires spatial resolution of less than 200 μm. Recently, a non-invasive method for the assessment of bone microarchitecture at the distal radius and tibia using high-resolution peripheral quantitative CT (HR-pQCT) has become available.

The aim of this study was to investigate the associations of patient characteristics, BMD [measured by dual-energy X-ray absorptiometry (DXA) and High-Resolution peripheral quantitative CT (HR-pQCT)], bone microarchitecture and calculated bone strength with secondary displacement of a DRF based on radiographic alignment parameters.

## Methods

### Study population

This cohort comprised patients aged 50–90 years presenting with a radiologically confirmed DRF, between November 2013 and June 2016. All consecutive patients were referred to the Fracture Liaison Service (FLS) and included in this study if they attended the FLS. After exclusion of patients with high-energy trauma (as this study focuses on fall-related fractures), osteomyelitis and bone metastasis, 251 patients with a recent DRF were included in this cross-sectional cohort study.

**Fig. 1:**
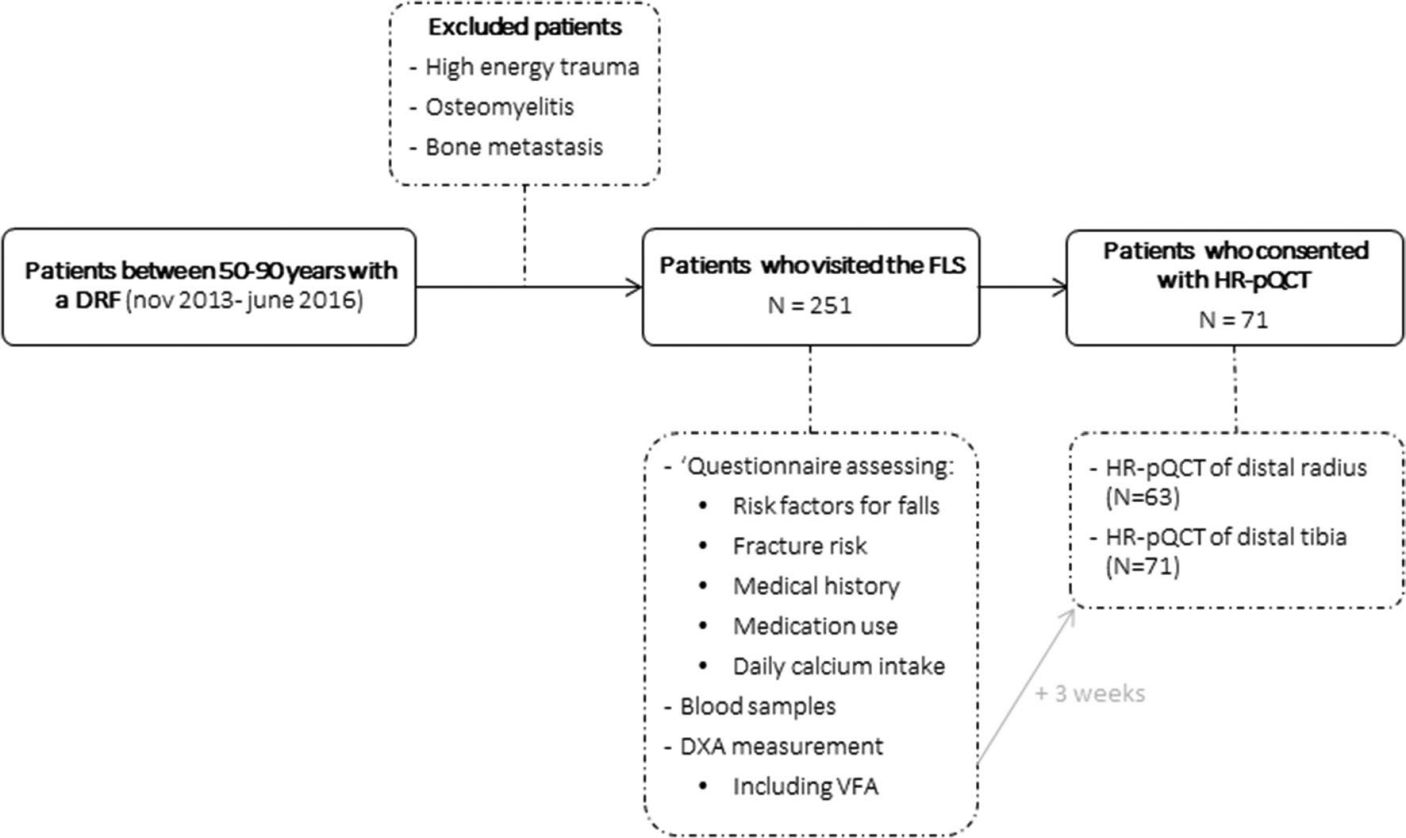
Flowchart of patients included in this study and corresponding investigations

At the FLS, patients received a detailed evaluation according to the Dutch guideline for treatment of osteoporosis. The evaluation consisted of a questionnaire assessing risk factors for falls, fracture risk, medical history including medication use, and daily dietary calcium intake. Additionally, blood samples were collected to identify metabolic disorders and a DXA measurement with vertebral fractures assessment (VFA) was performed 3–4 months after trauma (Fig. 1). If indicated, anti-osteoporosis treatment or treatment of newly diagnosed metabolic bone disorders was initiated according to current guidelines [12].

Of the 251 patients with a DRF included in this study, 71 participated in an observational 3-year follow-up study at the FLS (“Prospective evaluation of bone strength, physical activity, falls, subsequent fractures and mortality in patients presenting with a recent clinical fracture”). Approval was obtained from an institutional Review Board prior to performing the study (METC NL 45707.072.13). In that study, patients consented with HR-pQCT measurements of the distal radius (*N* = 63) and tibia (*N* = 71) and the baseline data are used for the HR-pQCT part of the current study. HR-pQCT measurements were conducted approximately 3 weeks after the DXA scan was performed (Fig. 1).

### DRF position and classification

**Fig. 2:**
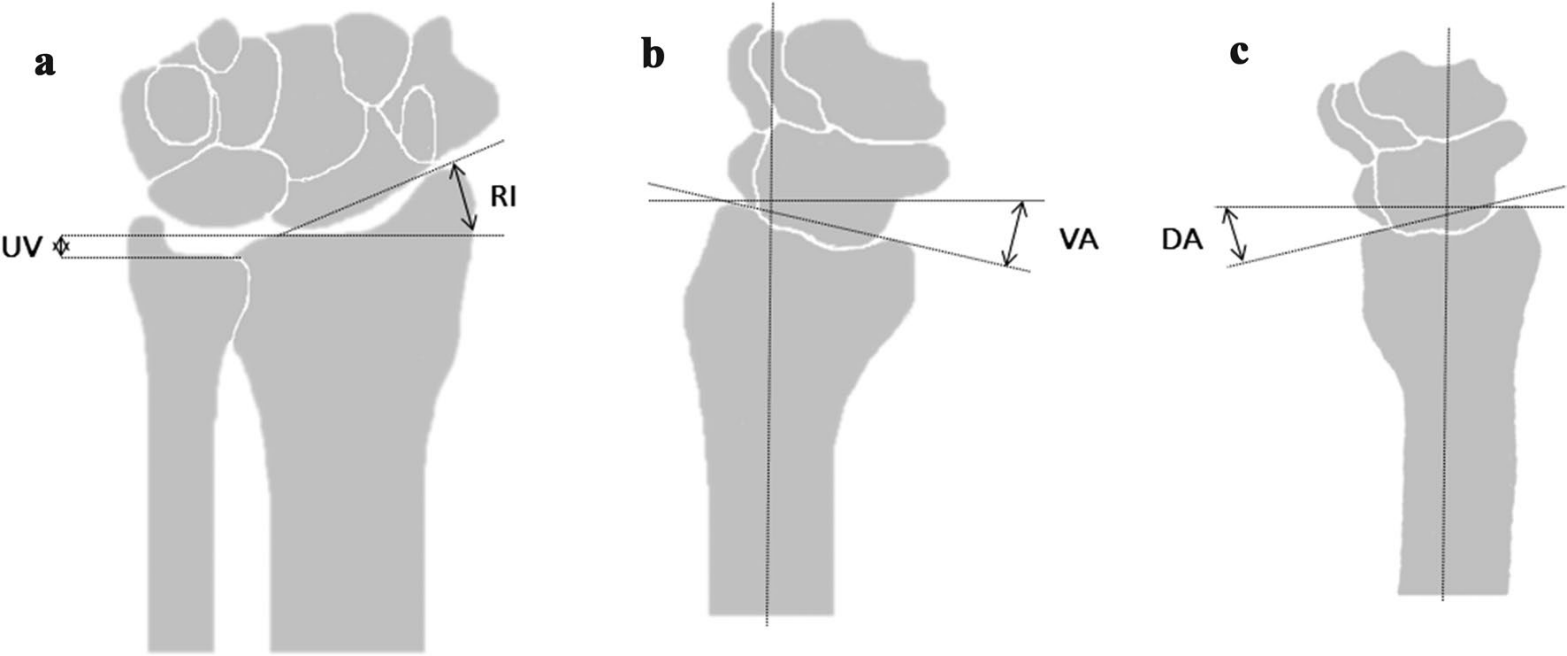
**a** Radiographic alignment parameters (RI/UV) to assess the distal radius [48]. **b** Radiographic alignment parameters (VA) to assess the distal radius [48]. **c** Radiographic alignment parameters (DA) to assess the distal radius [48]

**Fig. 3:**
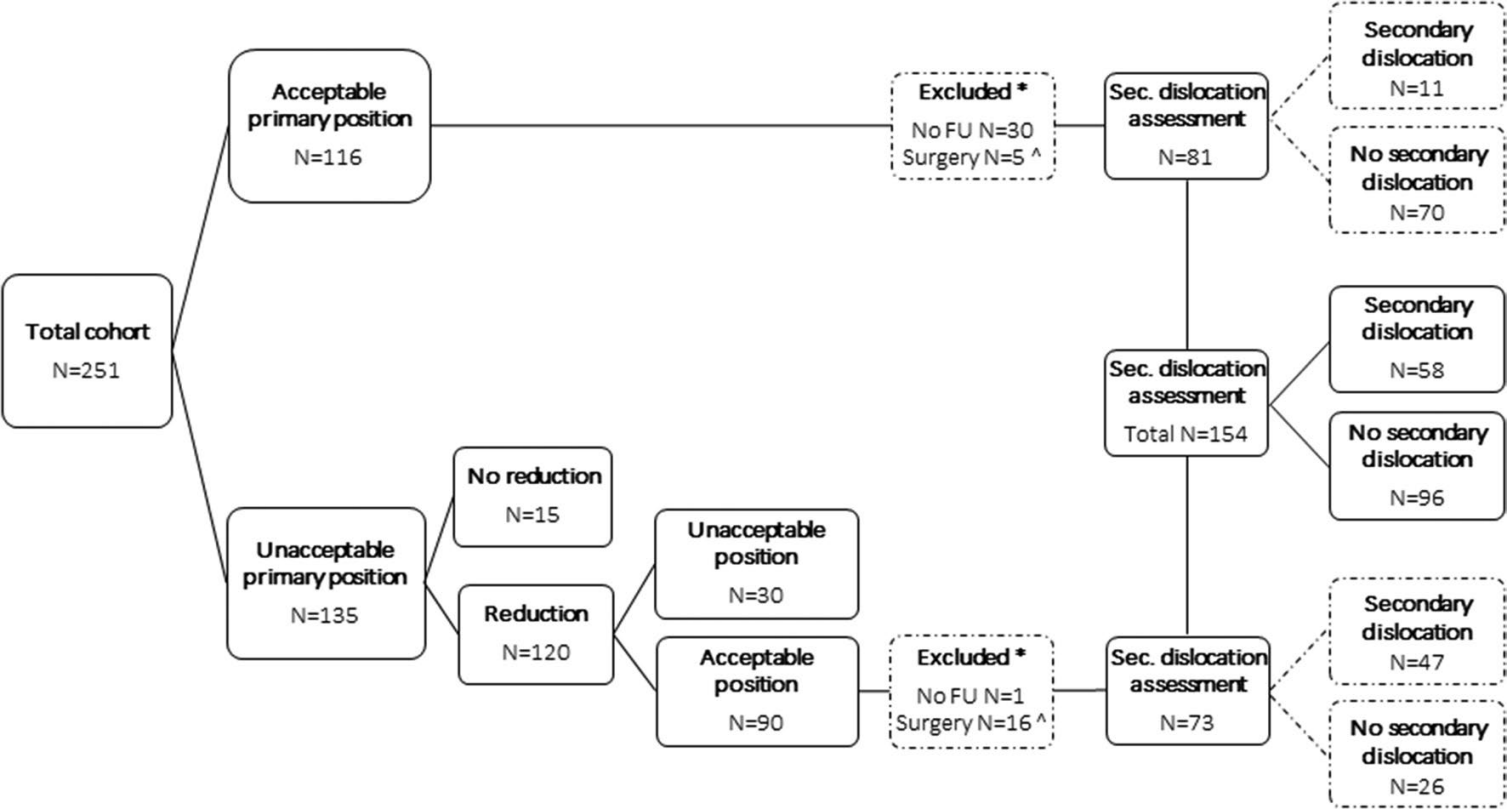
Flowchart of patient distribution and (eligibility for) secondary fracture displacement assessment

All conventional radiographs (antero-posterior and lateral) were used for assessment of alignment parameters, comprising angulation (dorsal = DA, volar/palmar = VA), radial inclination (RI) and ulnar variance (UV). DA/VA is measured on the lateral radiograph and represents the angle between a line perpendicular to the longitudinal axis of the radius and a line along the articular surface of the distal radius. RI is measured on the antero-posterior radiograph and represents the angle between a line connecting the tip of the radial styloid and the most ulnar point of the distal radius and a second line perpendicular to the longitudinal axis of the radius. UV represents the length of the ulna compared to the radius. According to the Dutch Guideline, DRFs were classified as fractures with an ‘unacceptable position’ when at least one of the following criteria was met; DA > 15°, VA > 20°, RI ≤ 15° and UV > 5 mm (Fig. 2a–c) [13]. Position at presentation, referred to as primary position, was assessed on the first radiographs of every patient. If reduction was applied the position was reassessed on radiographs following reduction, this position is referred to as baseline position. Adequate reduction was defined as regaining an acceptable position according to the criteria. In unreduced fractures with repeated radiographs immediately after cast immobilisation, baseline position is the position as measured on the repeated radiographs. In unreduced fractures without repeated radiographs baseline position was equivalent to primary position. All subsequent radiographs were assessed individually and used for secondary fracture displacement assessment, starting at baseline position. Patients with primary surgical intervention, an unacceptable baseline position or without follow-up radiographs were excluded from secondary fracture displacement assessment (Fig. 3). Secondary fracture displacement was defined as a displacement of the DRF that resulted in an unacceptable position, after an adequate baseline position in non-operatively treated patients.

All fractures were classified based on the AO/OTA classification on baseline plain radiographs by two independent investigators. DRFs were classified into three main types, namely type A (extra-articular), type B (partial articular) and type C (complete articular). Assessment by a third independent investigator was necessary for 51 patients with discrepant initial classification and resulted in agreement on another 47 fractures. For the remaining four fractures, conformity was reached by all three investigators in a consensus meeting.

### DXA and VFA

Two-dimensional BMD was measured at the lumbar spine (LS; L1–L4), total hip (TH) and femoral neck (FN) using DXA (Hologic QDR 4500, Hologic Inc., Bedford, MA, USA). Areal BMD measurements (g/cm^2^), were categorized according to the WHO criteria based on the lowest *T*-score at the LS, TH or FN into normal BMD (*T*-score ≥ − 1), osteopenia (*T*-score between − 1 and − 2.5) and osteoporosis (*T*-score ≤ − 2.5) [14].

VFA was performed on the DXA lateral spine images using quantitative morphometric assessment of vertebral height. The method described by Genant et al. was used to classify the severity of vertebral fractures (VFs); grade 1 (mild fracture, with vertebral height loss of 20–25%), grade 2 (moderate fracture with height loss of 25–40%) and grade 3 (severe fracture with height loss > 40%) [15].

### HR-pQCT

The second-generation HR-pQCT (XtremeCT II; Scanco Medical AG, Brüttisellen, Switzerland) was used to scan the contralateral radius and ipsilateral tibia. Scans were conducted and evaluated according to the standard protocol of the manufacturer (effective energy of 68 kVp, tube current of 1470 µA and 43 ms integration time) [16, 17]. The reference line was placed on the joint surface of the distal radius and tibia. The area to be scanned starts 9.0 mm proximally to the reference line and ends 1.2 mm distally to the reference line. Motion-induced degradation of the images was graded according to the method of Pialat et al. [17]. Images were processed according to the manufacturer’s standard protocol. The following parameters were analyzed: total, trabecular and cortical bone area [cm^2^], volumetric bone mineral density (vBMD) for the total, trabecular and cortical compartment [mgHA/cm^3^], trabecular bone volume fraction, trabecular number [mm^−1^], trabecular thickness [mm], trabecular separation [mm], cortical thickness [mm], cortical perimeter [mm], cortical porosity [%] and cortical pore diameter [mm]. In addition, micro-finite element analyses (micro-FEA) were generated by directly converting bone voxels in the segmented image to brick elements [18, 19]. Elements were assigned a Young’s modulus of 10 GPa and a Poisson’s ratio of 0.3 and for each model, four tests were simulated [20]. The first load case represented a ‘high friction’ compression test with a prescribed displacement in the axial direction of 1% of the total length, from which the compression stiffness [kN/mm] as well as the estimated strength was calculated [21–23]. The second load case represented a prescribed rotation of 0.01 rad around the longitudinal axis from which the torsional stiffness [kNmm/rad] was calculated. A third and fourth load case represented a prescribed rotation of 0.01 rad applied around the sagittal and transversal axes, respectively, thus inducing a state of pure bending in two directions, from which the bending stiffness in each direction was calculated. These four load cases were included to test if the fracture type is associated with a reduced stiffness in a specific loading direction.

### Statistical analysis

Data were analyzed using IBM SPSS Statistics, version 24 (IBM Corporation 1989, 2016). Normal distribution was tested with Q–Q plots and Kolmogorov–Smirnov analysis. Depending on the distribution, data are presented as mean with standard deviation (SD) or median with interquartile range (IQR). Chi-square tests were used to analyze differences in patient characteristics between the groups. Independent samples *t* tests were used to compare HR-pQCT parameters between patients with an acceptable and unacceptable primary position and between patients with and without secondary fracture displacement. Logistic regression analysis was used to investigate the independent association between secondary fracture displacement (yes vs. no) and HR-pQCT parameters. Bivariate analyses were conducted for primary reduction and all standardized scores (*z*-scores) of the HR-pQCT parameters for both the HR-pQCT radius group (*N* = 30) and HR-pQCT tibia group (*N* = 36). The cutoff value for significance to assess parameters in a multivariable model was defined as *p* ≤ 0.10. Due to the small sample size, multivariable analyses with adjustment for age and primary reduction were conducted separately for each significant HR-pQCT parameter in bivariate analysis. Receiver operating characteristic (ROC) analyses with area under the curve (AUC) measurements were conducted for all significant variables in the HR-pQCT analyses. Adjustment for age was conducted because of the potential effect on bone microarchitecture and strength. Significance level was set as *α* = 0.05.

## Results

### Primary position

Of 251 patients, 38 men (15%) and 213 women (85%) with a mean age of 67 years (SD ± 9), 116 (46%) had an acceptable and 135 (54%) an unacceptable primary position. One patients had a fracture with 20 degrees volar angulation and was therefore subjected to surgery. None of the fractures with volar angulation exceeded the range of 20 degrees. Patients with a DRF with primary unacceptable position were significantly older (69 vs. 66 years, *p* = 0.015) and had a lower body weight (68.6 vs. 73.5 kg, *p* = 0.035) and BMI (25.5 vs. 27.7 kg/m^2^, *p* = 0.048) than DRF patients with acceptable position [Table S-1]. The proportion of AO/OTA type A and B fractures was higher and type C fractures was lower in patients with an acceptable primary position compared to patients with an unacceptable primary position (*p* < 0.001). There was no difference in gender, BMD, number and severity of VFs, alcohol intake, smoking, calcium intake and vitamin D levels. Neither was there a difference in bone microarchitecture and strength measured by HR-pQCT between patients with a DRF with primary acceptable (tibia *N* = 31, radius *N* = 26) and unacceptable position (tibia *N* = 40, radius *N* = 35) [data not shown]. HR-pQCT scans of the distal tibia were conducted in all patients (*N* = 71), while distal radius scans could not be conducted in eight of these patients due to the presence of a bilateral DRF at the time of the study or a prior DRF at the contralateral side. Furthermore, two patients with unacceptable primary position had a bad-quality HR-pQCT of the distal radius and were therefore not included in HR-pQCT analysis.

### Secondary fracture displacement

Reduction was conducted in 120/135 patients with a primary unacceptable position with a success rate of 75%. This resulted in 206 patients with an acceptable baseline position. In 20 patients, surgery was conducted because of comminution of the fracture or patients’ preference. One patient had a fracture with 20 degrees volar angulation and, therefore, underwent surgery. After exclusion of these 21 patients and 31 patients without follow-up, 154 were eligible and assessed for secondary fracture displacement. Median follow-up with radiographs was 35 days (interquartile range 45 days), with numerous follow-up visits in this time range (Fig. 3). Secondary displacement occurred within the first 2 weeks in 39 patients (67%), in the third week in 12 patients (21%) and seven patients (12%) had a DRF displacement after 1 month. When comparing patients with secondary fracture displacement (*N* = 58) to those without (*N* = 96), we found no differences for age, gender distribution, BMI, BMD, number and severity of VFs, smoking, alcohol intake, calcium intake and vitamin D levels (Table 1). Primary reduction was significantly associated with secondary DRF displacement [OR 12.53 (95% CI 4.60–34.09), *p* < 0.001] in 154 patients.

**Table 1:** Characteristics of 154 patients with and without secondary fracture dislocation

	No secondary dislocation *N* = 96	Secondary dislocation *N* = 58	*p*-value
Female	83 (87)	54 (93)	0.202
Age (year)*	66 [14]	68.5 [13]	0.054
Weight (kg)*	73.3 [22.8]	66.6 [22.0]	0.101
Height (m)	1.63 ± 0.08	1.62 ± 0.06	0.340
BMI (kg/m^2^)*	26.4 [6.9]	25.4 [7.4]	0.174
AO			
A	57 (59.4)	36 (62.1)	0.334
B	14 (14.6)	4 (6.9)	
C	25 (26.0)	18 (31.0)	
BMI category			
< 30 (non obees)	63 (72.4)	40 (76.9)	0.557
≥ 30 (obees)	24 (27.6)	12 (23.1)	
Bone densitometry			
Normal BMD	16 (16.7)	8 (13.8)	0.710
Osteopenia	48 (50.0)	27 (46.6)	
Osteoporosis	32 (33.4)	23 (39.7)	
VFA			
No VF	87 (90.6)	51 (87.9)	0.595
≥ 1 Grade 2/3 VF	9 (9.4)	7 (12.1)	
Smoking			
Never	40 (42.6)	23 (41.1)	0.689
Past smoker	42 (44.7)	23 (41.1)	
Current smoker	12 (12.8)	10 (17.9)	
Alcohol intake			
< 1 unit/day	28 (30.4)	21 (39.6)	0.260
≥ 1 unit/day	64 (69.6)	32 (60.4)	
Calcium intake (mg/day)*	797 [396]	843 [428]	0.968
25-OH Vitamin D (nmol/l)			
< 30 (deficiency)	8 (8.3)	9 (15.5)	0.308
30–50 (insufficiency)	32 (33.3)	15 (25.9)	
> 50 (sufficiency)	56 (58.3)	34 (58.6)	

*BMI* body mass index, *BMD* bone mineral density, *VF* vertebral fracture

Data missing: length (14), weight (14), calcium intake (4), alcohol use (9), smoking (4)

Normally distributed data are presented as mean (SD)

Non normally distributed data * are presented as median [IQR]

HR-pQCT tibia and radius scans were available in 36 (23%), respectively, 30 (20%) patients for evaluation of secondary fracture displacement. Characteristics of patients with HR-pQCT measurement (*N* = 36) were not different compared to those without HR-pQCT (*N* = 118). Neither was there a difference in the proportion of secondary vs. non secondary dislocated fractures (Table 2).

**Table 2:** Characteristics of patients assessed for secondary dislocation with and without HR-pQCT measurement (*N* = 154)

	With HR-pQCT *N* = 36	Without HR-pQCT *N* = 118	*p*-value
Secondary dislocation	12 (33)	24 (24)	0.540
Female	32 (89)	105 (89)	0.597
Age (year)*	68 [12]	67 [16]	0.673
Weight (kg)*	71.5 [21]	69.3 [21]	0.981
Height (m)	1.65 ± 0.06	1.63 ± 0.07	0.118
BMI*	25.4 [5.0]	26.4 [8.4]	0.476
AO			
A	24 (66.7)	69 (58.5)	0.648
B	4 (11.1)	14 (11.9)	
C	8 (22.2)	35 (29.7)	
BMI category			
< 30 (non obees)	27 (81.8)	76 (71.7)	0.247
≥ 30 (obees)	6 (18.2)	30 (28.3)	
Bone densitometry			
Normal BMD	6 (16.7)	18 (15.3)	0.939
Osteopenia	18 (50.0)	57 (48.3)	
Osteoporosis	12 (33.3)	43 (36.3)	
VFA			
No VF	31 (86.1)	107 (90.7)	0.532
≥ 1 Grade 2/3 VF	5 (13.9)	11 (9.3)	
Smoking			
Never	14 (38.9)	49 (43.0)	0.863
Past smoker	17 (47.2)	48 (42.1)	
Current smoker	5 (13.9)	17 (14.9)	
Alcohol use			
< 1 unit/day	8 (22.2)	41 (37.6)	0.090
≥ 1 unit/day	28 (77.8)	68 (62.4)	
Calcium intake (mg/day)*	855 [379]	780 [404]	0.205
25-OH Vitamin D (nmol/l)			
< 30 (deficiency)	2 (5.6)	15 (12.7)	0.256
30–50 (insufficiency)	9 (25.0)	38 (32.2)	
> 50 (sufficiency)	25 (69.4)	65 (55.1)	

*BMI* body mass index, *BMD* bone mineral density, *VF* vertebral fracture

Data missing: length (14), weight (14), calcium intake (4), alcohol use (9), smoking (4)

Normally distributed data are presented as mean (SD). Non normally distributed data * as median [IQR]

At the distal radius (measured in 30 patients), total [OR 0.27 (95% CI 0.10–0.73), *p* = 0.010] and cortical [OR 0.31 (95% CI 0.12–0.80), *p* = 0.016] vBMD and cortical thickness [0.32 (95% CI 0.13–0.80), *p* = 0.015] were significantly lower in patients with secondary dislocated fractures. There were no differences for trabecular parameters and micro-FEA. The strongest determinant for secondary fracture displacement was primary reduction [OR 22.00 (95% CI 2.27–212.86), *p* = 0.008]. After adjustment for primary reduction, total [0.16 (95% CI 0.04–0.68), *p* = 0.013] and cortical [0.19 (95% CI 0.05–0.80), *p* = 0.024] vBMD and cortical thickness [0.13 (95% CI 0.02–0.74), *p* = 0.021] were significantly associated with secondary fracture displacement. Adjustment for age did not change the association of HR-pQCT parameters with secondary displacement of a DRF [Table S-2].

At the distal tibia (measured in 36 patients), total vBMD [OR 0.35 (95% CI 0.13–0.92), *p* = 0.034] and cortical pore diameter [OR 0.37 (95% CI 0.14–0.94), *p* = 0.036] at the distal tibia were significantly lower in patients with secondary dislocated fractures, while there were no differences for trabecular parameters and micro-FEA. After adjustment for primary reduction, both total vBMD [OR 0.06 (95% CI 0.01–0.64), *p* = 0.020] and cortical thickness [OR 0.22 (95% CI 0.06–0.84), *p* = 0.027] were significantly associated with secondary fracture displacement. After adjustment for age, none of the HR-pQCT parameters at the distal tibia was significantly associated with secondary displacement of a DRF [Table S-3].

## Discussion

DRFs with an unacceptable position are generally reduced at the ED as the first step of treatment. Recovery of alignment is thought to be important to preserve adequate function [1, 24]. Our study showed that fractures with an unacceptable position at need for reduction are, after adequate reduction, at high risk for secondary displacement. In addition, this study shows for the first time, that secondary fracture displacement was independently associated with lower total and cortical vBMD and lower cortical thickness at the distal radius, measured by HR-pQCT, after adjustment for primary reduction while no other clinical parameter including BMD, vertebral fractures was associated with secondary fracture displacement. The HR-pQCT results are clinically relevant as the odds of secondary fracture displacement is 81–87% higher in patients with lower total and cortical vBMD and cortical thickness at the distal radius.

Previous data regarding timing of displacement are controversial. Some studies reported that all DRFs dislocate in the first two weeks, whereas others describe it as a gradual process [6, 25, 26]. Our data indicate that 88% of all displacements occur in the first 3 weeks. Although nearly significant (*p* = 0.054), our data did not confirm previous findings that secondary fracture displacement was associated with age [5–8]. This might be due to the age limit of 50–90 years in our study, whereas Abbaszadegan et al. included patients from the age of 18 years. Gender, BMI, VFA, smoking/alcohol status, calcium intake and vitamin D levels were not associated with secondary displacement of a DRF in our study.

The association between BMD, measured by DXA, and the ability to maintain adequate position of a DRF has previously been investigated. Clayton et al. (2009) concluded that osteoporosis (T-score < − 2.5) was associated with secondary DRF displacement in a cohort of 137 patients aged over 55 [27]. On the contrary, Robin et al. (2014) studied patients aged over 65 years with a displaced DRF and concluded that there was no relationship between BMD, measured by DXA, and the ability to maintain position after adequate reduction [28]. These findings are in line with our study where we observed no difference in or association with BMD, measured by DXA, between patients with and without secondary fracture displacement.

Although cortical integrity is widely suggested to play a role in DRF stability [5, 6, 29, 30], assessment of cortical comminution is frequently conducted without a clear definition [5, 9]. For example, dorsal comminution is assessed on conventional radiographs, but exact measurement is not possible due to limited resolution. A non-invasive method recently available for the assessment of bone microarchitecture at the extremities is HR-pQCT [23, 31, 32]. In our study, secondary fracture displacement was independently associated with lower total and cortical vBMD and lower cortical thickness at the distal radius, measured by HR-pQCT. At the distal tibia, lower total vBMD and lower cortical thickness appeared to be determinants for secondary DRF displacement, however, after adjustment for age, these HR-pQCT parameters were no longer associated with secondary DRF displacement. Although the HR-pQCT measurements were performed in a limited number of patients we found significant associations with total and cortical vBMD and cortical thickness at the distal radius. We believe that the comparison of the affected with the unaffected distal radius is the most appropriate way to study the associations of skeletal parameters with secondary fracture displacement. To best of our knowledge, this is the first study demonstrating the association of bone microarchitecture with secondary DRF displacement.

Some issues were limiting in our study. First, closed reduction was performed by the treating physician at the ED by which some variability might exist in the decision, the technique and quality (as result of experience level) of reduction. Second, due to the retrospective design of our study, radiographic alignment parameters were only assessed at the fractured radius. Since both wrists of one individual can be considered as symmetrical [33], van Eerten et al. [34] assessed the implementation of a technique comparing the fractured site with the unaffected side. They conclude that only reproducibility of radial inclination measurement, and not of radial length or dorsal/volar angulation, improved after implementation of the new template technique. Third, fracture assessment in this study was conducted using radiographs since previous literature shows no significant increase of inter and intra observer agreement of the AO classification of DRFs when using CT [35]. However, recent studies suggests that additional CT scanning may be of importance for the accuracy of scoring the fracture types [36, 37]. Fourth, for classification of the complexity of DRFs, the AO/OTA classification was used. This is one of many available classification systems being the Frykman, Fernandez, Melone and universal classification system [35, 38, 39], unfortunately none of these systems has perfect reproducibility rates [35, 40–42]. In contrast to other classification systems, the AO/OTA classification has a strong intra- and inter-observer reliability for assessment of the main type (A—extra articular, B—partially articular, C—complete articular). Classifying the AO subtype is not recommended based on the poor intra- and inter-observer reliability [43]. In concordance with this, consensus rate in our study for two independent investigators was 79.7%. Furthermore, classification of main type was in line with previous published papers [44, 45]. Fourth, since assessment of BMD and VFs was only possible in FLS attenders, we studied a selected cohort of patients presenting at the ED with a DRF. Due to the retrospective design of our study, not all patients underwent HR-pQCT. However, there was no difference between patients with and without HR-pQCT measurement and no difference in secondary fracture displacement distribution between the total cohort and the subgroup with HR-pQCT measurement. Accordingly, the study results of the subset of patients with HR-pQCT are representative for and can be extrapolated to our total cohort of patients with a DRF. Finally, as described in the result section, 14 patients with an unacceptable position were treated non-operatively. This was due to the fact that they were not willing or suitable, as judged by their treating physician, to undergo surgery. It is well founded not to subject older patients with multiple comorbidities to manipulation/surgery since it is proven to be of minimal value [46, 47], however, this might have caused bias in our study.

In conclusion, our data demonstrate that the most important determinant for secondary displacement of a DRF was primary reduction. However, while other patient characteristics, BMD and VF status were not associated with secondary fracture displacement, lower total and cortical vBMD and lower cortical thickness at the distal radius were independently associated with secondary displacement of a DRF. This indicates that besides primary reduction, cortical bone quality may be important for the risk of secondary displacement of DRFs.

## Electronic supplementary material

Below is the link to the electronic supplementary material.

Supplementary file1 (Docx 17 kb)

Supplementary file2 (Docx 17 kb)

Supplementary file3 (Docx 17 kb)
