# Supplementary material for: Association of secondary displacement of distal radius fractures with cortical bone quality at the distal radius
Source: Arch Orthop Trauma Surg. 2020 Oct 31;141(11):1909–18. doi: 10.1007/s00402-020-03658-2 (PMC8497288; doi:10.1007/s00402-020-03658-2)
Supplement: Supplementary file 1 — Supplementary file1 (Docx 17 kb) [file 402_2020_3658_MOESM1_ESM.docx]

**Table S-1.** Characteristics of 251 patients with an acceptable and unacceptable DRF position

|  | **Primary position unacceptable** N = 135 | **Primary position acceptable** N = 116 | p-value |
| --- | --- | --- | --- |
| Female | 117 (87) | 96 (83) | *N.S.* |
| Age (y)* | 69 [13] | 66 [14] | 0.015 |
| Weight (kg)* | 68.6 [19.1] | 73.5 [23.7] | 0.035 |
| Height (m) | 1.63 ± 0.10 | 1.63 ± 0.09 | *N.S.* |
| BMI (kg/m2)* | 25.5 [6.2] | 27.7 [7.7] | 0.048 |
|  |  |  |  |
| AO |  |  | 0.000 |
| A | 61 (45.2) | 70 (60.3) |  |
| B | 12 (8.9) | 24 (20.7) |  |
| C | 62 (45.9) | 22 (19.0) |  |
|  |  |  |  |
| BMI category |  |  | *N.S.* |
| <30 (non obees) | 95 (80.5) | 70 (66.0) |  |
| ≥30 (obees) | 23 (19.5) | 36 (34.0) |  |
|  |  |  |  |
| Bone densitometry |  |  | *N.S.* |
| Normal BMD | 18 (13.3) | 20 (17.2) |  |
| Osteopenia | 64 (47.4) | 56 (48.3) |  |
| Osteoporosis | 53 (39.3) | 40 (34.5) |  |
|  |  |  |  |
| VFA |  |  | *N.S.* |
| No VF | 116 (85.9) | 105 (90.5) |  |
| ≥ 1Grade 2/3 VF | 19 (14.1) | 11 (9.5) |  |
|  |  |  |  |
| Smoking |  |  | *N.S.* |
| Never | 58 (43.9) | 49 (43.3) |  |
| Past smoker | 54 (40.9) | 50 (44.2) |  |
| Current smoker | 20 (15.2) | 14 (12.4) |  |
|  |  |  |  |
| Alcohol use |  |  | *N.S.* |
| < 1 unit/day | 41 (32.5) | 35 (32.4) |  |
| ≥ 1 unit/day | 85 (67.5) | 73 (67.6) |  |
|  |  |  |  |
| Calcium intake (mg/day)* | 813 [390] | 770 [420] | *N.S.* |
|  |  |  |  |
| 25-OH Vitamin D(nmol/l) |  |  | *N.S.* |
| <30 (deficiency) | 13 (9.6) | 13 (11.2) |  |
| 30-50 (insufficiency) | 43 (31.9) | 31 (26.7) |  |
| >50 (sufficiency) | 79 (58.5) | 72 (62.1) |  |

**Legends table S-1.**

BMI = body mass index. BMD = bone mineral density. VF= vertebral fracture.

Data missing: length (26), weight (26), calcium intake (6), alcohol use (9), smoking (6).

Normally distributed data are presented as mean (SD). Non normally distributed data * as median [IQR]
