# Supplementary material for: Association of secondary displacement of distal radius fractures with cortical bone quality at the distal radius
Source: Arch Orthop Trauma Surg. 2020 Oct 31;141(11):1909–18. doi: 10.1007/s00402-020-03658-2 (PMC8497288; doi:10.1007/s00402-020-03658-2)
Supplement: Supplementary file 2 — Supplementary file2 (Docx 17 kb) [file 402_2020_3658_MOESM2_ESM.docx]

**Table S-2.** Associations of bone micro-architecture and strength with secondary fracture dislocation (vs. no secondary fracture dislocation) in 30 patients with HR-pQCT at the distal radius.

|  | **OR (CI)**  Unadjusted | **p-value** | **OR (CI)**  Age adjusted | **p-value** | **OR (CI)**  Primary reduction adjusted | **p-value** |
| --- | --- | --- | --- | --- | --- | --- |
| Primary reduction | 22.00 [2.27-212.86] | 0.008 | - | - | - | - |
| Total area | 1.44 [0.65-3.15] | 0.376 | - | *N.S.* | - | *N.S.* |
| Trabecular area | 2.06 [ 0.89-4.77] | 0.092 | 1.96 [0.81-4.79] | *N.S.* | 3.97 [1.00-15.69] | *N.S.* |
| Cortical area | 0.46 [0.18-1.18] | 0.108 | - | *N.S.* | - | *N.S.* |
| Total vBMD | 0.27 [0.10-0.73] | 0.010 | 0.31 [0.11-0.85] | 0.023 | 0.16 [0.04-0.68]*^ | 0.013 |
| Trabecular vBMD | 0.55 [0.22-1.33] | 0.182 | - | *N.S.* | - | *N.S.* |
| Cortical vBMD | 0.31 [0.12-0.80] | 0.016 | 0.37 [0.13-0.99] | 0.049 | 0.19 [0.05-0.80]*^ | 0.024 |
| Trabecular BV fraction | 0.61 [0.24-1.53] | 0.291 | - | *N.S.* | - | *N.S.* |
| Trabecular thickness | 1.70 [0.69-4.21] | 0.248 | - | *N.S.* | - | *N.S.* |
| Trabecular separation | 1.90 [0.89-4.06] | 0.100 | 1.75 [0.71-4.32] | *N.S.* | 1.92 [0.70-5.27] | *N.S.* |
| Cortical perimeter | 1.80 [0.77-4.17] | 0.173 | - | *N.S.* | - | *N.S.* |
| Cortical porosity | 0.63 [0.26-1.54] | 0.309 | - | *N.S.* | - | *N.S.* |
| Cortical thickness | 0.32 [0.13-0.80] | 0.015 | 0.39 [0.15-0.99] | 0.050 | 0.13 [0.02-0.74]*^ | 0.021 |
| Cortical pore diameter | 0.54 [0.20-1.44] | 0.215 | - | *N.S.* | - | *N.S.* |
| Torsion stiffness | 0.82 [0.33-2.06] | 0.679 | - | *N.S.* | - | *N.S.* |
| Compression stiffness | 0.39 [0.13-1.16] | 0.090 | 0.48 [0.16-1.42] | *N.S.* | 0.32 [0.09-1.18] | *N.S.* |
| Compression ultimate force | 0.39 [0.13-1.19] | 0.098 | 0.48 [0.16-1.46] | *N.S.* | 0.32 [0.09-1.18] | *N.S.* |
| Bending stiffness horizontal | 0.67 [0.26-1.72] | 0.409 | - | *N.S.* | - | *N.S.* |
| Bending stiffness vertical | 0.95 [0.36-2.50] | 0.913 | - | *N.S.* | - | *N.S.* |

**Legends table S-2**

OR=odds ratio for secondary fracture dislocation vs no secondary fracture dislocation. ORs for HR-pQCT parameters are presented per standard deviation (SD).

vBMD = volumetric bone mineral density. BV = bone volume. N.S. = not significant.

* OR of primary reduction in multivariable model:
- with total vBMD: OR 71.96 [2.80-1848.20], p 0.010
- with cortical vBMD: OR 62.92 [2.20-1803.45], p 0.016
- with cortical thickness: OR 123.29 [2.82-5388.05], p 0.012

^ AUC of significant HR-pQCT parameters in primary reduction adjusted model;

- Total vBMD; AUC = 0.824, 95% CI 0.67-0.98, p 0.003

- Cortical vBMD; AUC = 0.819, 95% CI 0.67-0.97, p 0.003

- Cortical thickness; AUC = 0.787, 95 % CI 0.61-0.96, p 0.009
