# Supplementary material for: Association of secondary displacement of distal radius fractures with cortical bone quality at the distal radius
Source: Arch Orthop Trauma Surg. 2020 Oct 31;141(11):1909–18. doi: 10.1007/s00402-020-03658-2 (PMC8497288; doi:10.1007/s00402-020-03658-2)
Supplement: Supplementary file 3 — Supplementary file3 (Docx 17 kb) [file 402_2020_3658_MOESM3_ESM.docx]

**Table S-3.** Associations of bone micro-architecture and strength with secondary fracture dislocation (vs. no secondary fracture dislocation) in 36 patients with HR-pQCT at the distal tibia.

|  | **OR (CI)**  Unadjusted | **p-value** | **OR (CI)**  Age adjusted | **p-value** | **OR (CI)**  Primary reduction adjusted | **p-value** |
| --- | --- | --- | --- | --- | --- | --- |
| Primary reduction | 18.33 [2.02-166.73] | 0.010 | - | - | - | - |
| Total area | 1.20 [0.62-2.31] | 0.585 | - | *N.S.* | - | *N.S.* |
| Trabecular area | 1.32 [0.69-2.55] | 0.406 | - | *N.S.* | - | *N.S.* |
| Cortical area | 0.48 [0.18-1.27] | 0.137 | - | *N.S.* | - | *N.S.* |
| Total vBMD | 0.35 [0.13-0.92] | 0.034 | 0.40 [0.15-1.05] | 0.063 | 0.06 [0.01-0.64]* | 0.020 |
| Trabecular vBMD | 0.54 [0.24-1.23] | 0.141 | - | *N.S.* | - | *N.S.* |
| Cortical vBMD | 0.52 [0.24-1.09] | 0.085 | 0.64 [0.28-1.45] | 0.286 | 0.41 [0.16-1.04] | *N.S.* |
| Trabecular BV fraction | 0.55 [0.24-1.26] | 0.157 | - | *N.S.* | - | *N.S.* |
| Trabecular thickness | 1.03 [0.51-2.10] | 0.926 | - | *N.S.* | - | *N.S.* |
| Trabecular separation | 1.00 [0.54-1.86] | 0.990 | - | *N.S.* | - | *N.S.* |
| Cortical perimeter | 1.32 [0.67-2.61] | 0.424 | - | *N.S.* | - | *N.S.* |
| Cortical porosity | 0.96 [0.49-1.91] | 0.918 | - | *N.S.* | - | *N.S.* |
| Cortical thickness | 0.44 [0.18-1.12] | 0.085 | 0.58 [0.23-1.46] | 0.248 | 0.22 [0.06-0.84]* | 0.027 |
| Cortical pore diameter | 0.37 [0.14-0.94] | 0.036 | 0.48 [0.20-1.20] | 0.117 | 0.38 [0.14-1.09] | *N.S.* |
| Torsion stiffness | 0.82 [0.36-1.86] | 0.630 | - | *N.S.* | - | *N.S.* |
| Compression stiffness | 0.55 [0.22-1.37] | 0.196 | - | *N.S.* | - | *N.S.* |
| Compression ultimate force | 0.68 [0.30-1.53] | 0.345 | - | *N.S.* | - | *N.S* |
| Bending stiffness horizontal | 0.91 [0.43-1.93] | 0.813 | - | *N.S.* | - | *N.S.* |
| Bending stiffness vertical | 0.79 [0.35-1.81] | 0.578 | - | *N.S.* | - | *N.S.* |

**Legends table S-3**

OR=odds ratio for secondary fracture dislocation vs no secondary fracture dislocation. ORs for HR-pQCT parameters are presented per standard deviation (SD).

vBMD = volumetric bone mineral density. BV = bone volume. N.S. = not significant.

* OR of primary reduction in multivariable model:
- with total vBMD: 251.94 [3.90-16274.94], p 0.009
- with cortical thickness: OR 46.34 [3.10-692.30], p 0.005

^ AUC of significant HR-pQCT parameters in primary reduction adjusted model;

- Total vBMD; AUC = 0.731, 95% CI 0.55-0.91, p 0.026

- Cortical thickness; AUC = 0.719, 95% CI 0.53-0.90, p 0.035
